# Association between abnormal uterine artery pulsatility index and the risk of fetal congenital heart defects: a hospital-based cohort study

**DOI:** 10.1038/s41598-023-50167-4

**Published:** 2023-12-21

**Authors:** Chen Zhu, Cheng-Jie Xu, Jiang-Nan Wu, Wei Zhao, Yan-Lai Hu, Ying Yao, Yun-Yun Ren

**Affiliations:** 1 Department of Ultrasound, Obstetrics and Gynecology Hospital of Fudan University, No. 588 Fangxie Road, Shanghai, China; 2 Department of Information Technology, Obstetrics and Gynecology Hospital of Fudan University, Shanghai, China; 3 Department of Clinical Epidemiology, Obstetrics and Gynecology Hospital, Fudan University, Shanghai, China

**Keywords:** Ultrasonography, Pregnancy outcome

## Abstract

To explore the associations between high uterine artery pulsatility index (UtA-PI) values and congenital heart disease (CHD) risk and whether they differed between singleton and multiple pregnancies. This hospital-based cohort study involving 52,047 pregnant women who underwent prenatal examinations from 2012 to 2016. Infants born to the included pregnant women were followed until 42 days after birth to identify those with CHDs. Generalized estimating equations were used to estimate the associations of high right UtA-PI (> 95th percentile) values with maternal preeclampsia and fetal CHDs. Logistic regression analyses were conducted using path analysis models to quantify the effect of high right UtA-PI values on fetal CHD risk. A total of 42,552 women and 43,470 infants (147 with CHDs) were included. Preeclampsia risk was associated with a high right UtA-PI in singleton-pregnant women (adjusted PR, 3.01; 95% CI 2.57–3.52). CHD risk was marginally associated with a high right UtA-PI in singleton-pregnant women (adjusted PR, 2.26, 95% CI 1.03–4.95). Considering only two factors, 96.0% of the fetal CHD risk was mediated by preeclampsia in singleton-pregnant women, while 93.8% of the risk was related to a high right UtA-PI in multiple-pregnant women. A high right UtA-PI was marginally associated with an increased fetal CHD risk in singleton-pregnant women and might play an important role in multiple-pregnant women. Further studies are warranted to confirm these findings given the high loss to follow-up rate.

## Introduction

Congenital heart defects (CHDs) are the most common anomalies in infants, affecting 8 of 1000 live births worldwide^1^. CHDs are a major cause of morbidity and mortality^2^. Since the mechanism of CHDs is still unknown, researchers tend to identify factors and biomarkers associated with CHDs to better understand their origins^3–6^.

Biomarkers associated with angiogenic imbalance, such as proangiogenic signaling proteins (e.g., vascular endothelial growth factor and placental growth factor) and antiangiogenic proteins (e.g., soluble endoglin and fms-like tyrosine kinase 1), are associated with CHDs^6^ and are abnormally altered in the early stages of preeclampsia (PE)^3,7,8^. Previous studies from different countries have linked maternal PE and infantile CHDs and considered angiogenic mechanisms as a common pathway for both conditions^4,5,9,10^. Although a strong association between maternal PE and fetal CHDs across pregnancies was found by Boyd and colleagues^5^, which might support a maternal origin of the disease^11^, the underlying mechanism and direction of the association remain unclear.

The uterine artery pulsatility index (UtA-PI), as well as some biomarkers of angiogenic imbalance, such as serum pregnancy-associated plasma protein-A and placental growth factor, are markers of placental perfusion and function^12^ and are used to screen for maternal PE^13^ because the related persistence of high-resistance maternal uterine circulation, impaired placental perfusion, and maternal antiangiogenic stress response are all involved in the pathogenesis of PE^14^. However, the effect of placental perfusion and function on fetal CHDs is still controversial, and knowledge of this effect is limited to singleton pregnancies^12^. The extent of the effects of maternal and placental origin factors on neonatal CHDs is still unknown. We therefore conducted a retrospective cohort study to determine the relationship between a high UtA-PI and the risk of infantile CHDs, to quantify the extent of the effect of maternal PE (presumed to be a maternal origin factor) and UtA-PI (a marker of placental perfusion) on the risk of infantile CHD development and to determine whether these effects vary by multiplicity.

## Method

### Study design and data collection

We conducted a hospital-based cohort study of pregnant women who underwent prenatal examinations at the hospital between April 2012 and August 2016 in Shanghai, China^15^. All women and infants born to them were followed up until the 42nd day after birth (the last case was completed in March 2017). Two clinical examinations and surveys were conducted after delivery and on the 42nd day of postpartum examination. Basic characteristics and UtA-PI measurements of the subjects and fetal CHD identification messages were collected by face-to-face interview or by physical or instrumental examination. Women who were lost to follow-up were excluded from the analysis. These excluded women had a lower likelihood of receiving a UtA-PI measurement than those who were included in the analysis (50.8% vs. 74.6%, P < 0.001); however, no difference in the proportion of women with a high right UtA-PI was found (4.7% vs. 4.9%, P = 0.53). As this was a retrospective study that only collected and utilized previous data, and did not involve subjects' privacy investigation or informed consent, the Ethics Committee of the Obstetrics and Gynecology Hospital of Fudan University approved the waiver of informed consent (No. 2017-35, 2017-35-C1). All methods explained herein were performed in accordance with relevant guidelines and regulations, and together with the study protocol were approved by the Ethics Committee of the Obstetrics and Gynecology Hospital of Fudan University (No. 2017-35, 2017-35-C1), and also got registered by the Chinese Clinical Trial Registry (No. ChiCTR1900028151).

### Right UtA-PI measurement

UtA-PI on both sides was measured using GE Voluson-E6 and GE Voluson-E8 ultrasound devices (GE Healthcare, Zipf, Austria) at 21^+0^ to 24^+6^ weeks of gestation according to standardized protocols^16^. There were differences in the left and right UtA-PIs and in their association with CHD (Table S1). In addition, prior analyses of the study indicated that no difference in the left UtA-PI was found between infants with and without CHD (Table S2). Thus, the right UtA-PI was selected for the analysis since different UtA indices have varied predictive values^17^. Women were classified into three groups: women with high or normal right UtA-PIs and women with UtA-PIs that were not available. High right UtA-PI was defined as right UtA-PI values > the 95th percentile by gestational week at the test since the distribution was not a normal distribution. Because the UtA test was optional, 11,048 women (25.4% of the total sample) without the measurement were classified as the missing UtA-PI group. Basic characteristics were unbalanced between women with and without the UtA-PI measurement, but no difference was found for the occurrence of CHD or PE (Table S3).

### CHD identification

CHDs were diagnosed by sonography or magnetic resonance imaging between delivery and discharge or were confirmed by the Shanghai Perinatal Statistics Collection System between discharge and the postpartum examination conducted on the 42nd day after birth^18^. Information on CHD cases diagnosed before delivery and from women who were lost to follow-up was not available in the present study. CHDs in the study included ventricular septal defect (VSD, Q21.001), atrial septal defect (ASD, Q21.102), tetralogy of Fallot (Q21.300), coarctation of the pulmonary artery (Q25.601), truncus arteriosus (Q25.001), common ventricle (Q20.401), dextrocardia (Q24.001), diverticula of the right atrium (Q24.811), patent foramen ovale (Q21.103), and transposition of the great arteries (Q20.301). Details of the CHD cases are displayed in Table S4.

### PE and potential confounders

PE is defined as the onset of hypertension and proteinuria after 20 weeks of gestation in women who were previously normotensive^19^. Pregnant women with PE were further classified into mild and severe groups according to the progress of the disease^19^. Patients with eclampsia and chronic hypertension with superimposed PE were included in the severe and mild PE categories since there were few cases in the study^4^. In addition, we considered gestational hypertension to be mild PE, as was done in a previous study^4^. Potential confounders in the present study included maternal age at delivery (≤ 24, 25–34, or ≥ 35 years), resident location (Shanghai or other provinces), parity (nulliparous or pluriparous), mode of conception (assisted or natural conception), gestational diabetes mellitus (yes or no), and multiplicity (singleton or multiple births). Gestational diabetes mellitus was diagnosed according to a 75-g oral glucose tolerance test conducted between 24 and 28 weeks of gestation^20^. Considering that the missing rates for each variable (except UtA-PI) were very low (ranging from 0 to 0.12%), we excluded infants born to pregnant women who had any missing data for the variables included in the analyses (0.14% of the births).

### Statistical analysis

The prevalence of CHDs and PE and proportions of covariates across the right UtA-PI groups were compared using the chi-square test or Fisher’s exact test. Considering the potential correlations between twins of the same pregnancy and the mechanisms of fetal CHD in singleton versus multiple pregnancies, adjusted prevalence ratios (PRs) and 95% confidence intervals (95% CIs) for CHDs (VSD, ASD, and any CHDs) and PE in the high right UtA-PI group were estimated relative to those of the normal right UtA-PI group by generalized estimating equation (GEE) models that included unbalanced basic characteristics. Models are run when there are at least 5 exposed cases. Log-binomial models with a Poisson distribution were used in the GEE models, as performed in a previous study^4^. In these models, women without right UtA-PI measurements were also included. However, the correlations in these women were not shown.

Logistic regression analyses using path analysis models were constructed to decompose the total risk of CHDs according to the effect of maternal PE and the effect of high right UtA-PI. Because high right UtA-PI measurements may overlap with the timing of the onset of PE and CHD, causal association determination is more difficult, and a common pathogenesis may exist (Fig. S1A). However, in the present study, to quantify the magnitude of their roles, we hypothesized that high right UtA-PI interacts with PE and that there is a difference in the common role in the onset of CHD (Fig. S1B). The factor (maternal PE or high right UtA-PI) associated significantly with CHDs was set as a direct factor, and another factor was set as an indirect factor^21^. The total effect of the direct factor on the risk of CHDs was decomposed into a direct effect on the risk of CHDs and an indirect (or interaction) effect on the risk of CHDs, which may be mediated by the association between direct and indirect factors. If both factors were significantly associated with CHDs, both were set as direct factors in sequence, and the results of the path analyses were then combined according to the weight of the coefficient to explain the extent of the effect of a high right UtA-PI or PE.

We conducted sensitivity analyses, in which we restricted the analysis to women who had a UtA-PI test to exclude potential bias induced by the exclusion of women with missing UtA-PIs. Path analysis models were performed with Stata 12 software (Statacorp, Texas, USA). All other statistical tests were conducted using IBM SPSS Statistics version 22.0 (IBM Corp., Armonk, NY, USA). A P value < 0.05 was considered to indicate statistical significance.

## Results

### Population characteristics and right UtA-PI distribution

**Figure 1:**
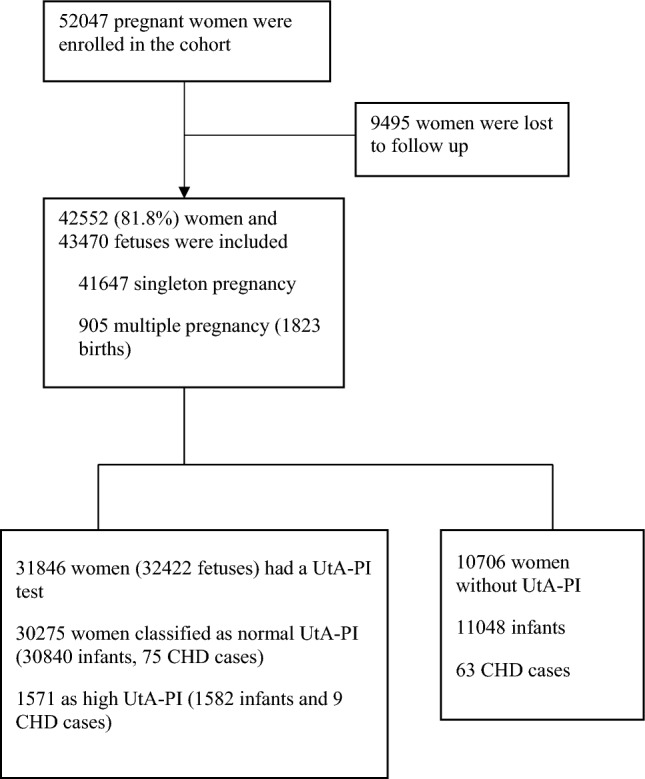
Flowchart of the study.

In the present cohort of 52,047 pregnant women, 42,552 (81.8%) were followed up until confirmation of the diagnosis of CHDs in the 43,470 infants born to them in May 2017, with an average follow-up of 22.0 weeks (range, 9.0–27.0; SD, 1.6). Among these women, 31,846 women (32,422 infants) had received the right UtA-PI measurement (Fig. 1). According to the gestational week-specific 95th percentiles (1.52, 1.42, 1.33, 1.31 and 1.35 for 20, 21, 22, 23 and 24 weeks of gestation, respectively), 4.9% of pregnancies (1571/31,846) were classified as the high right UtA-PI group. Basic characteristics (e.g., maternal age, residence, parity) were balanced between women with normal and high right UtA-PIs, except for the proportion of infants with assisted conception (2.9% vs. 1.6%, P = 0.003) (Table 1).

**Table 1:** Prevalence of congenital heart defects and preeclampsia and maternal characteristics based on groups of right uterine artery pulsatility index.

Outcomes and variables	Groups by right uterine artery pulsatility index^a^			P value for difference among the three groups	P value for difference between groups of ≤ P95 and > P95
	≤ P95 (infant no. = 30,840)	> P95 (infant no. = 1582)	Data missing (infant no. = 11,048)		
	No. (%)				
Congenital heart defect^b^					
Ventricular septal defect	18 (0.6)	2 (1.3)	26 (2.4)	< 0.001^§^	0.26^§^
Atrial septal defect	54 (1.8)	6 (3.8)	30 (2.7)	0.040^§^	0.07^§^
Any	75 (2.4)	9 (5.7)	63 (5.7)	< 0.001	0.02^§^
Preeclampsia					
Mild	1512 (4.9)	144 (9.1)	564 (5.1)	< 0.001	< 0.001
Severe	288 (0.9)	92 (5.8)	191 (1.7)	< 0.001	< 0.001
Any	1800 (5.8)	236 (14.9)	755 (6.8)	< 0.001	< 0.001
Maternal age at delivery (years)				< 0.001	0.79
< 25	1359 (4.4)	69 (4.4)	760 (6.9)		
25–34	26,696 (86.6)	1362 (86.1)	8827 (79.9)		
≥ 35	2785 (9.0)	151 (9.5)	1461 (13.2)		
Residence				< 0.001	0.51
Shanghai	24,405 (79.1)	1241 (78.4)	7625 (69.0)		
Other provinces	6435 (20.9)	341 (21.6)	3423 (31.0)		
Nulliparous	25,817 (83.7)	1334 (84.3)	8263 (74.8)	< 0.001	0.52
Gestational diabetes mellitus	2558 (8.3)	148 (9.4)	1103 (10.0)	< 0.001	0.14
Assisted conception	892 (2.9)	26 (1.6)	547 (5.0)	< 0.001	0.003
Multiple pregnancies	1126 (3.7)	22 (1.4)	675 (6.1)	< 0.001	< 0.001

^a^The P95 of the right UtA-PI was 1.52, 1.42, 1.33, 1.31 and 1.35 for 20, 21, 22, 23 and 24 weeks of gestation, respectively.

^b^Prevalence per 1000 infants.

^§^P value for Fisher’s exact tests.

### Prevalence of CHDs and PE

A total of 147 CHD cases were diagnosed, with an overall prevalence of CHD of 3.4 per 1000 infants (147 infants of 43,470 births). Among the 84 CHD cases identified in women who received a UtA-PI measurement, 75 cases were from mothers with a normal UtA-PI, and 9 were from women classified as having a high right UtA-PI (Fig. 1). The prevalence of total CHDs was higher among infants born to women with a high right UtA-PI than among those whose mother had a normal right UtA-PI (5.7‰ vs., 2.4‰, P = 0.021). However, no significant difference in the prevalence of VSD or ASD was found between the groups of women with high and normal right UtA-PIs (Table 1). A total of 2791 pregnant women (6.4%) were diagnosed with PE. The prevalence of PE was higher among pregnant women with a high right UtA-PI than among those with a normal right UtA-PI (14.9% vs. 5.8%, P < 0.001) (Table 1).

### High right UtA-PI and risk of fetal CHDs

Compared with pregnant women with normal right UtA-PIs, women with high right UtA-PIs had an increased risk of subsequent maternal PE with singleton pregnancies (adjusted PR, 3.01; 95% CI 2.57–3.52). Similar results were found for the relationship of high right UtA-PI with mild and severe PE (Table 2). The risk of fetal CHDs was associated with a high right UtA-PI in women with singleton pregnancies (PR, 1.92, 95% CI 0.87–4.27) (Table 2). If left or right UtA-PI > the 95th percentile is simultaneously considered classified as a PI abnormality, the association with fetal CHD was weakened (adjusted PR, 1.59, 95% CI 0.84–3.00) (Table S2). In women with multiple pregnancies, women with a high right UtA-PI were more likely to have a CHD baby than those with a normal right UtA-PI (2/18 vs. 20/1130; P = 0.045 for Fisher’s exact test). Sensitivity analyses indicated a marginal association (adjusted PR, 2.26, 95% CI 1.03–4.95) between a high right UtA-PI measurement and the risk of fetal CHD in women with singleton pregnancies (Table 3).

**Table 2:** Association of a high right uterine artery pulsatility index with the risk of infantile congenital heart defect and maternal preeclampsia.

Outcomes	Singleton births (N = 41,647)					Multiple births (N = 1823)				
	Normal right UtA-PI	High right UtA-PI	UtA-PI data missing	Prevalence ratio (95% CI)		Normal right UtA-PI	High right UtA-PI	UtA-PI data missing	Prevalence ratio (95% CI)	
	No. of infants			Unadjusted	Adjusted^a^	No. of infants			Unadjusted	Adjusted^a^
No CHDs	29,655	1553	10,343	Reference	Reference	1110	20	642	Reference	Reference
VSD	15	2	13	–	–	3	0	13	–	–
ASD	41	4	15	–	–	13	2	15	–	–
Any CHDs^b^	59	7	30	2.27 (1.03 to 4.97)	1.92 (0.87 to 4.27)	16	2	33	–	–
No PE	28,162	1332	9785	Reference	Reference	878	14	508	Reference	Reference
Mild PE	1344	136	464	2.14 (1.78 to 2.57)	2.07 (1.71 to 2.51)	168	8	100	2.99 (0.86 to 10.43)	2.37 (0.91 to 6.20)
Severe PE	208	92	124	9.35 (7.27 to 12.03)	6.68 (5.24 to 8.51)	80	0	67	–	–
Any PE	1552	228	588	3.11 (2.68 to 3.61)	3.01 (2.57 to 3.52)	248	8	167	2.02 (0.58 to 7.02)	1.56 (0.60 to 4.05)

^a^Models adjusted for maternal age at delivery (< 25, 25–34, or ≥ 35 years), residence (Shanghai or other provinces), parity (nulliparous or pluriparous), mode of conception (assisted or natural conception), and gestational diabetes mellitus (yes or no); maternal preeclampsia (no, mild or severe) was also included in the models for congenital heart defects.

^b^Models adjusted for maternal age at delivery (< 25, 25–34, or ≥ 35 years), residence (Shanghai or other provinces), parity (nulliparous or pluriparous), gestational diabetes mellitus (yes or no), and mode of conception (assisted or natural conception); maternal preeclampsia (no, mild or severe) was also included in the models for congenital heart defects.

The estimated PRs in the included women without right UtA-PI measurements are not shown.

**Table 3:** Sensitivity analyses restricted to women with the right UtA-PI measurement.

Outcomes	Women with the right UtA-PI measurement (N = 32,422)					
	Singleton births (N = 31,274)			Multiple births (N = 1148)		
	Normal right UtA-PI	High right UtA-PI	Adjusted PR (95% CI)	Normal right UtA-PI	High right UtA-PI	Adjusted PR (95% CI)
	No. of infants			No. of cases/total No		
No CHDs	29,655	1553	Reference	1110	20	Reference
VSD	15	2	–	3	0	–
ASD	41	4	–	13	2	–
Any CHDs	59	7	2.26 (1.03 to 4.95)	16	2	–
No PE	28,162	1332	Reference	878	14	Reference
Mild PE	1344	136	2.13 (1.77 to 2.57)	168	8	3.35 (1.38 to 8.16)
Severe PE	208	92	9.40 (7.31 to 12.10)	80	0	–
Any PE	1552	228	3.11 (2.68 to 3.61)	248	8	1.55 (0.60 to 4.04)

Models adjusted for maternal age at delivery (< 25, 25–34, or ≥ 35 years), residence (Shanghai or other provinces), parity (nulliparous or pluriparous), mode of conception (assisted or natural conception), and gestational diabetes mellitus (yes or no). Models are run and reported when there are at least 5 exposed cases.

### Association between maternal PE and fetal CHDs

The prevalence of CHD was higher in infants exposed to maternal PE than in those without the disorder (13.3‰ vs. 2.7‰, P < 0.001). Compared with infants not exposed to maternal PE, infants exposed to PE had an increased risk of CHDs (adjusted PR, 4.4; 95% CI 2.7–7.2) in women with singleton birth. The association remained for ASD (adjusted PR, 3.9; 95% CI 2.1–7.5) and VSD (adjusted PR, 3.7; 95% CI 1.5–9.4) (Table 4). No significant associations were found for women with multiple births (Table 4).

**Table 4:** Variants of preeclampsia and prevalence of congenital heart defects.

Congenital heart defects	Singleton births				Multiple births			
	No PE (n = 39,279)	Mild PE (n = 1944)	Severe PE (n = 424)	Any PE (n = 2368)	No PE (n = 1400)	Mild PE (n = 276)	Severe PE (n = 147)	Any PE (n = 423)
Ventricular septal defect								
No. of cases (‰)	24 (0.6)	1 (0.5)	5 (11.8)	6 (2.5)	14 (10.0)	2 (7.2)	0 (0.0)	2 (4.7)
Adjusted PR (95% CI)^a^	1.0		16.8 (6.1 to 46.4)	3.7 (1.5 to 9.4)	1.0			
Atrial septal defect								
No. of cases (‰)	48 (1.2)	4 (2.1)	8 (18.9)	12 (5.1)	19 (13.6)	7 (25.4)	4 (27.2)	11 (26.0)
Adjusted PR (95% CI)	1.0		14.2 (6.6 to 30.4)	3.9 (2.1 to 7.5)	1.0	1.7 (0.5 to 5.1)		1.9 (0.7 to 5.0)
Any CHDs								
No. of cases (‰)	75 (1.9)	6 (3.1)	15 (35.4)	21 (8.9)	35 (25.0)	11 (39.9)	5 (34.0)	16 (37.8)
Adjusted PR (95% CI)	1.0	1.5 (0.7 to 3.5)	17.2 (9.7 to 30.6)	4.4 (2.7 to 7.2)	1.0	1.6 (0.7 to 4.0)	1.4 (0.4 to 4.8)	1.6 (0.7 to 3.5)

^a^Models adjusted for maternal age at delivery (< 25, 25–34, or ≥ 35 years), residence (Shanghai or other provinces), parity (nulliparous or pluriparous), mode of conception (assisted or natural conception), and gestational diabetes mellitus (yes or no). Models are run and reported when there are at least 5 exposed cases.

### Role of high right UtA-PI and maternal PE in the risk of fetal CHDs

In singleton pregnancies, path analyses indicated that maternal PE was associated with an increased likelihood of CHDs (total effect: adjusted PR, 4.48, 95% CI 2.64–7.61), in which 96.0% of the overall effect was mediated/interacted by high right UtA-PI (indirect effect: adjusted PR, 1.06, 95% CI 0.97–1.17). In contrast, a high right UtA-PI dominated the risk of CHDs in women with multiple births, and only 6.2% of the effect of a high right UtA-PI and maternal PE on the risk of infantile CHDs was mediated/interacted by maternal PE (Table 5).

**Table 5:** Decomposition of the total effect of a high right uterine artery pulsatility index (or preeclampsia) on the odds of congenital heart defects into a direct and an indirect effect mediated through preeclampsia (or a high right uterine artery pulsatility index).

	Total effect		Direct effect			Indirect effect			Estimated size of the effect of preeclampsia on the risk of infantile congenital heart defects (%)
	Coef.	Prevalence ratio (95% CI)	Direct factor	Coef.	Prevalence ratio (95% CI)	Indirect factor	Coef.	Prevalence ratio (95% CI)	
Singletons	1.50	4.48 (2.64 to 7.61)	PE	1.44	4.22 (2.48 to 7.18)	High PI	0.06	1.06 (0.97 to 1.17)	96.0
Multiples	1.94	6.96 (1.97 to 24.50)	High PI	1.82	6.17 (1.55 to 24.29)	PE	0.12	1.13 (0.88 to 1.45)	6.2

Adjusted for maternal age at delivery (< 25, 25–34, or ≥ 35 years), residence (Shanghai or other provinces), parity (nulliparous or pluriparous), mode of conception (assisted or natural conception), and gestational diabetes mellitus (yes or no).

According to the results, only one path analysis was conducted, in which the effect of PE was selected as the direct factor in women with singleton pregnancies, while the effect of the high right UtA-PI was set as a direct factor among those with multiple pregnancies.

## Discussion

In the present cohort study, we found that first, women with a high right UtA-PI were marginally associated with the risk of fetal CHDs in singleton pregnancies and had a higher risk of CHDs in multiple pregnancies. Second, taking the two factors into account, maternal PE has a greater impact on the risk of fetal CHDs in singleton births, while a high maternal right UtA-PI was predominantly responsible for fetal CHDs in multiple pregnancies. These findings support different pathophysiological mechanisms for fetal CHDs between singleton and multiple pregnancies and suggest the clinical value of the right UtA-PI measurement in screening for fetal CHD.

Consistent with the findings of previous studies^4,5^, we identified the association between maternal PE and fetal CHDs among singleton pregnancies. Compared with maternal PE, quantitative analysis indicated that only 4% of the total effect of maternal PE on the risk of fetal CHDs was attributed to a high maternal right UtA-PI. These findings provide further evidence for a predominantly maternal origin effect for the development of CHD in singleton pregnancies^4,5,12^. Possible mechanisms for the association involve angiogenic imbalances initiated at the start of pregnancy and endothelial/placental dysfunction related to the etiology of maternal PE^4,5,12,22^. The association between maternal PE and CHDs was affected by the severity of maternal PE, as found in previous studies in which maternal PE was classified into early- and late-onset PE^4,10^. Severe maternal PE yielded significantly strong associations with CHDs in singleton pregnancies, while no significant correlations were found between mild maternal PE and CHDs in the present study. This heterogeneity may be attributed to the homogeneous conditions between early- and late-onset PE^10,23^. Severe PE, such as early-onset PE, is associated with impaired placentation, whereas mild PE is considered a manifestation of underlying metabolic insulin resistance. Furthermore, maternal PE was not significantly associated with the risk of CHDs in multiple pregnancies. The difference between singleton and multiple pregnancies might be attributed to maternal PE being a heterogeneous disorder in multiple pregnancies^24^, in which elevated fms-like tyrosine kinase 1 levels are due to increased placental mass rather than pathologic overexpression and may not pose as a great risk for CHDs as maternal PE in singleton pregnancies^4^.

Placental dysfunction was recently found to be related to CHD and some specific malformations (e.g., male hypospadias)^25^. In recent studies, Graupner et al.^26^ demonstrated that UtA-PI revealed a significant increase in all CHD subgroups, and it worsened toward the end of pregnancy, and Ruiz et al.^27^ drew a similar conclusion: 61% of CHD cases suggested a certain degree of placental impairment (mean UtA-PI above the 95th percentile). In our study, among multiple births, fetuses exposed to a high right UtA-PI had a significantly higher risk of CHDs. The underlying mechanisms are still unclear. The uterus undergoes dextrorotation during pregnancy, which makes the right uterine artery travel more lateral to the uterus relative to the left uterine artery^28^, and may be more indicative of inadequate perfusion. The pathology of fetal CHDs in multiple pregnancies may differ from that among singleton pregnancies and is more likely to be affected by placental perfusion and function.

Twin pregnancies are at increased risk for numerous fetal and maternal complications that are often related to impaired uteroplacental function^29^. Impaired uteroplacental circulation plays a central role in the pathogenesis of neonatal complications^30^. Measurement of impedance to flow in the uterine arteries, such as the measurement of the UtA-PI, may reflect the progress of placentation and be a useful predictor of uteroplacental complications^24^. A high UtA-PI may be a consequent manifestation of defective or absent remodeling of the myometrial segment of the uteroplacental arteries^30,31^ and accompanied by low serum PAPP-A and placental growth factor (PlGF) that are not conducive to the formation of the fetal cardiovascular system^22,32^, which were found in the group with CHDs but not in the control subjects in a prospective screening study^12^. Furthermore, the classification of the P95 of the right UtA-PI values by gestational weeks of the total sample (> and ≤ P95) may improve the predictive accuracy of a high right UtA-PI for fetal CHDs since the right UtA-PI values in twin pregnancies were lower than those in singleton pregnancies^29^.

A high right UtA-PI was associated with the risk of maternal PE, which is in accordance with the results of previous studies^31,33^. Impaired trophoblastic invasion of the spiral arteries and their conversion from high-impedance narrow vessels to wide nonmuscular channels and impaired placental perfusion and hypoxia stimulate the release of inflammatory factors that cause endothelial cell activation and generalized vasoconstriction, which may link high UtA-PI and risk of PE^34^.

There are some limitations in the present study. First, although the low prevalence of CHDs may be partially attributed to the free folic acid supplement program for pregnant women that has been implemented since 2009 in China^35^, the prevalence of CHDs in the present study might be underestimated since women are at a higher risk of giving birth to CHD babies, such as mothers with miscarriages and terminated pregnancies^36^,were not included. Furthermore, information on the prenatal diagnosis of CHD was not available. All cases were isolated forms and nonisolated forms, and some rare and potentially missed CHD types might not be observed because the sample size was not large enough. The diagnosis of CHDs was limited to the period between delivery and the 42nd day postpartum examination, and there was a high rate of loss to follow-up. Therefore, these shortages might affect the representativeness of fetal CHD in the present study and result in a potential bias. However, we do not think this shortage may hugely affect the strong associations (e.g., severe PE and risk of CHD) found in the present study. In addition, due to the small number of CHD cases, we did not estimate the strength of some associations, such as the association between a high right UtA-PI and the risk of fetal CHDs in multiple-pregnant women. However, the decomposition analysis was still estimated, so this result should be interpreted cautiously. Second, although the sample size was relatively large and the unbalanced basic characteristics between women with and without the UtA-PI measurement were included in the multivariable models, this was a single-center study, and we were not able to rule out all potential bias, which may then limit the generalizability of the findings. More importantly, UtA-PI data were missing for 25% of the subjects in this cohort, and although our sensitivity analyses showed that the results were not affected, the possibility of potential selection bias in the study could not be ruled out. Third, some known risk factors for neonatal CHD^37^, such as congenital infections, exposure to medications (e.g., anticonvulsants, recreational drugs, and family history of CHD)and maternal BMI, as well as potential confounders for PE and elevated UtA PI (e.g., use of aspirin and gestational thyroid function^20^), were not available in the present study due to the study design. The absence of these factors might result in a potential overestimation of the association. Fourth, we acknowledged that the robustness of the results might be affected since only 9 CHD cases were included in the analyses in the high right UtA-PI group. Finally, many inherited and noninherited factors are associated with CHD, in which mutations in regulators of heart development during embryogenesis are the major underlying mechanisms^38^. However, in our study, only two factors (maternal PE and high right UtA-PI) were used in the quantitative analysis, which might result in an overestimation of the maternal original effect for singleton pregnancies and of the placental perfusion effect for multiple pregnancies.

In addition, although we measured data on bilateral UtA-PIs, we selected the right UtA-PI as the study variable because of the differences in its association with CHD as well as the simultaneous consideration that left and right UtA-PIs as a basis for categorization would weaken the association of the right UtA-PI with CHD. Although the results might point out the direction of association and enhance the clinical actionability of the right UtA-PI as a potential screening indicator, the results might be limited to comparing the relative importance of the two factors in CHD risk. Based on these deficiencies, the robustness of the results may be compromised. Therefore, further prospective cohort studies with large sample sizes are warranted to address these concerns.

## Conclusions

In conclusion, in this large sample cohort study of pregnant Chinese women, maternal PE, especially severe maternal PE, was associated with a high right UtA-PI measured during the second trimester and with the risk of fetal CHDs in women with singleton pregnancies. A high right UtA-PI was correlated with the risk of fetal CHDs in women with multiple pregnancies. Taking only two factors into account, maternal original effects are predominantly responsible for the development of fetal CHDs in women with singleton pregnancies, whereas fetal CHDs born to women with multiple pregnancies are mainly related to placental perfusion and function. Given the study limitations, the results should be considered with caution, and further research is needed to validate the findings.

### Supplementary Information

Supplementary Information.

## Data Availability

The data that support the findings of this study are available from the corresponding author upon reasonable request.
