# Supplementary material for: Association between abnormal uterine artery pulsatility index and the risk of fetal congenital heart defects: a hospital-based cohort study
Source: Sci Rep. 2023 Dec 21;13:22924. doi: 10.1038/s41598-023-50167-4 (PMC10739791; doi:10.1038/s41598-023-50167-4)
Supplement: Supplementary file 1 — Supplementary Information. [file 41598_2023_50167_MOESM1_ESM.pdf]

Association between abnormal uterine artery pulsatility index and the risk of fetal congenital heart defects: a hospital-based cohort study

Chen Zhu, Cheng-Jie Xu, Jiang-Nan Wu, Wei Zhao, Yan-Lai Hu, Ying Yao, Yun-Yun Ren

Table S1. Difference between right and left UtA-PI and its association with risk of CHDs.

Table S2. UtA-PI measurements in infants with and without congenital heart defects.

Table S3. The outcomes and basic characteristics between women with and without the UtA-PI measurement.

Table S4. Details of the CHD cases.

Figure S1. Hypothetical relationship diagram in the decomposition analyses.

Table S1. Difference between right and left UtA-PI and its association with risk of CHDs.

PI	*Mean*	*SD*	*CHD*	
			*PR (95% CI)**	*P value*
Right	*0.835*	*0.28*	*2.11 (1.14-3.91)*	*0.018*
Left	*0.861*	*0.29*	*1.01 (0.48-2.11)*	*0.99*
Difference (95% CI)	*-0.025 (-0.029 to -0.022).*			

* *Adjusted for maternal age at delivery (<25, 25-34, or ⩾ 35 years), residence (Shanghai or other provinces), parity (nulliparous or pluriparous), mode of conception (assisted or natural conception), gestational diabetes mellitus (yes or no), and multiplicity (singleton or multiple pregnancies).*

Table S2. UtA-PI measurements in infants with and without congenital heart defects.

UtA-PI		*Congenital heart defects*				
		Yes	*No*	*P value*	*Adjusted PR (95%CI)*	*P value*
Right PI, continuous variable		*0.89(0.37)*	*0.83(0.28)*	*0.05*		
Right, category variable				*0.037*		
	≤P95	*75*	*30765*		*Reference*	
	>P95	*9*	*1573*		*(1.22-4.88)*	*0.018*
Left PI, continuous variable		*0.85 (0.33)*	*0.86(0.29)*	*0.71*		
Left, category variable				*0.24*		
	≤P95	*79*	*30724*		*Reference*	
	>P95	*5*	*1571*		*1.23 (0.50-3.04)*	*0.50*

The adjusted PR was 1.59 (0.84-3.00) for CHD using high PI defined as the right or left was >P95.

Table S3. The outcomes and basic characteristics between women with and without the UtA-PI measurement.

Outcomes or characteristics	Singleton pregnancy (N=41647)			Multiple pregnancy (N=905)		
	UtA-PI measurement		P value	UtA-PI measurement		P value
	Yes (N=31274)	No (N=10373)		Yes (N=572)	No (N=333)	
CHD (n, per 1000 infants)	66 (2.1)	30 (2.9)	0.15	10 (0.2)	14 (0.4)	0.05
PE (n, %)	1780 (5.7)	588 (5.7)	0.93	127 (22.2)	84 (25.2)	0.30
Maternal age (years) (n, %)			<0.001			<0.001
< 25	1396 (4.5)	714 (6.9)		16 (2.8)	22 (6.6)	
25-34	27080 (86.6)	8328 (80.3)		487 (85.1)	246 (73.8)	
⩾ 35	2798 (8.9)	1331 (12.8)		69 (12.1)	65 (19.5)	
Shanghai resident (n. %)	24807(79.3)	7223 (69.6)	<0.001	418 (73.1)	200 (60.1)	<0.001
Nulliparous (n,%)	27102 (86.7)	8239 (79.4)	<0.001	25 (4.4)	12 (3.6)	0.57
Assisted conception (n,%)	500 (1.6)	253 (2.4)	<0.001	207 (36.2)	146 (43.8)	0.023

Table S4. Details of the CHD cases.

CHD types	*No. of cases*	*Proportion (%)*	*Prevalence, per 1000 infants,(95%CI)*
Atrial septal defect	*80*	*54.42*	*2.1 (1.6-2.5)*
Ventricular septal defect	*42*	*28.57*	*1.1 (0.8-1.4)*
Tetralogy of Fallot	*3*	*2.04*	*0.6 (0.4-0.8)*
Coarctation of the pulmonary artery	*5*	*3.40*	
Complicated defects	*2*	*1.36*	
Patent foramen ovale	*1*	*0.68*	
Transposition of the great arteries	*3*	*2.04*	
Patent ductus arteriosus	*11*	*7.48*	

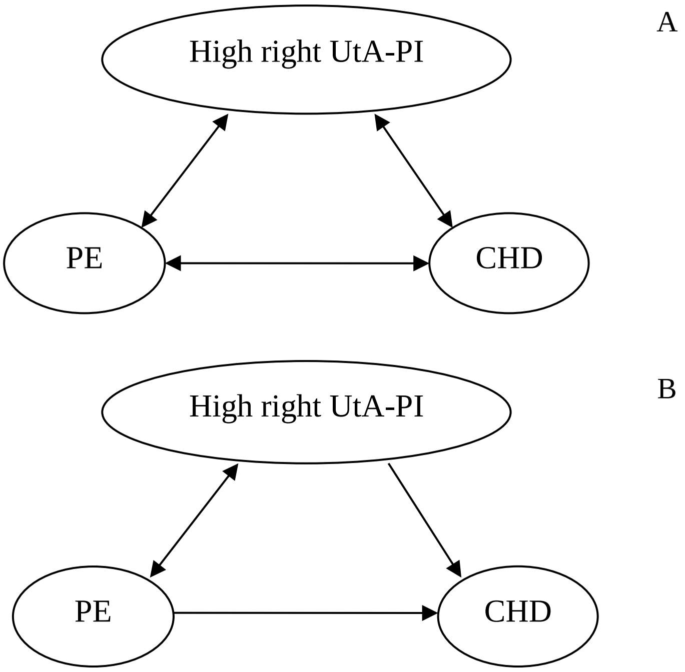

Figure S1. Hypothetical relationship diagram in the decomposition analyses.

A. Possible correlation paths; B. Hypothetical paths in the study.
